# Efficacy and safety of reduced-dose daratumumab plus bortezomib and dexamethasone (DVd-lite) in newly diagnosed MGRS patients

**DOI:** 10.3389/fimmu.2026.1722204

**Published:** 2026-06-23

**Authors:** Huan Xie, Maoyuan Xiang, Meijuan Zhang, Fanqiao Meng, Yan Qi, Kehong Chen, Dongfeng Zeng

**Affiliations:** 1 Department of Hematology, Daping Hospital, Army Medical University, Chongqing, China; 2 Department of Nephrology, Daping Hospital, Army Medical University, Chongqing, China

**Keywords:** bortezomib, clonal-directed therapy, daratumumab, MGRS, proteinuria

## Abstract

**Background:**

Clone-directed therapy is essential for managing monoclonal gammopathy of renal significance (MGRS), as it reduces the nephrotoxic monoclonal protein burden and preserves renal function. However, due to the rarity and frequent misdiagnosis of MGRS, there is no evidence-based standard first-line treatment for MGRS.

**Methods:**

This open-label, single-arm, prospective clinical trial assessed the efficacy and safety of the combination of reduced-dose daratumumab (Dara), bortezomib, and dexamethasone (DVd-lite) in ten newly diagnosed MGRS patients. The treatment protocol consisted of six cycles of the DVd-lite regimen, which included Dara (8–10 mg/kg biweekly), bortezomib (1.3 mg/m^2^ weekly) and dexamethasone (5–20 mg weekly).

**Results:**

The median follow-up duration of this cohort was 13.1 months (range: 4.7-17.6 months). The results showed a hematological overall response rate (ORR) of 80% and a kidney ORR of 90%. Moreover, the complete response rates in hematology and kidney were both 60%. In addition, only one patient experienced a grade 3 treatment-related adverse event.

**Conclusions:**

These findings demonstrated that the DVd-lite regimen is an effective and well-tolerated first-line treatment option for newly diagnosed MGRS patients.

**Clinical Trial Registration:**

https://www.chictr.org.cn/, identifier ChiCTR2400081273.

## 1 Introduction

Monoclonal gammopathy of renal significance (MGRS) encompasses a spectrum of kidney disorders caused by nephrotoxic monoclonal immunoglobulin (MIg) secreted by clonal plasma cells or B-cells (1, 2). Because of the low burden of clonal cells, MGRS does not meet the diagnostic criteria for multiple myeloma or B-cell malignancies (3, 4). Nevertheless, the toxic MIg secreted by these clonal cells can persistently cause kidney damage, ultimately leading to end-stage renal disease (1). Consequently, clonal-directed therapy is required to reduce nephrotoxic MIg load, thereby preserving kidney function.

Currently, there is no evidence-based recommended first-line treatment for MGRS, and clinical guidelines primarily outline general therapeutic principles and enumerate potential treatment options rather than recommending standardized regimens (1, 5–7). Daratumumab (Dara), an anti-CD38 monoclonal antibody, has shown hematological efficacy and renal response in some clinical studies involving light chain (AL) amyloidosis, light chain deposition disease (LCDD), and proliferative glomerulonephritis with monoclonal immunoglobulin deposition (PGNMID) (8–10). Furthermore, a retrospective study revealed that Dara combined with bortezomib achieved a significantly higher hematological response rate compared to Dara monotherapy in MGRS patients (11). However, because MGRS patients usually exhibit water and sodium retention secondary to cardiac and/or renal insufficiency, long-term use of full-dose Dara might exacerbate volume overload and compromise organ function (7). In addition, recurrent infection is a major clinical problem during full-dose Dara therapy, potentially adversely impacting patient survival outcomes (12, 13). More broadly, recent hematologic immunotherapy studies have highlighted the importance of incorporating safety-optimization strategies when potent immune-based treatments are associated with infection, immune dysregulation, or other clinically relevant toxicities (14, 15). Therefore, developing dose-optimized combination regimens that maintain efficacy while minimizing toxicity is clinically imperative to improve outcomes in MGRS patients.

Given the typically low plasma cell burden in MGRS and the aforementioned safety concerns, we proposed that a reduced-dose Dara combined with bortezomib and dexamethasone (DVd-lite) regimen might be a more suitable therapeutic strategy for MGRS patients. Based on this rationale, we initiated an open-label, single-arm, prospective clinical trial to evaluate the efficacy and safety profile of the DVd-lite regimen in newly diagnosed MGRS patients.

## 2 Study design

This clinical trial was conducted at Daping Hospital, Army Medical University (Chongqing, China) from February 2024 to August 2025 (ChiCTR2400081273). The trial was conducted in accordance with the principles of the Declaration of Helsinki and the International Conference on Harmonisation Guidelines for Good Clinical Practice, with prior approval of the institutional review board and the ethics committee of the local hospital. All patients provided written informed consent prior to participation.

**Figure 1:**
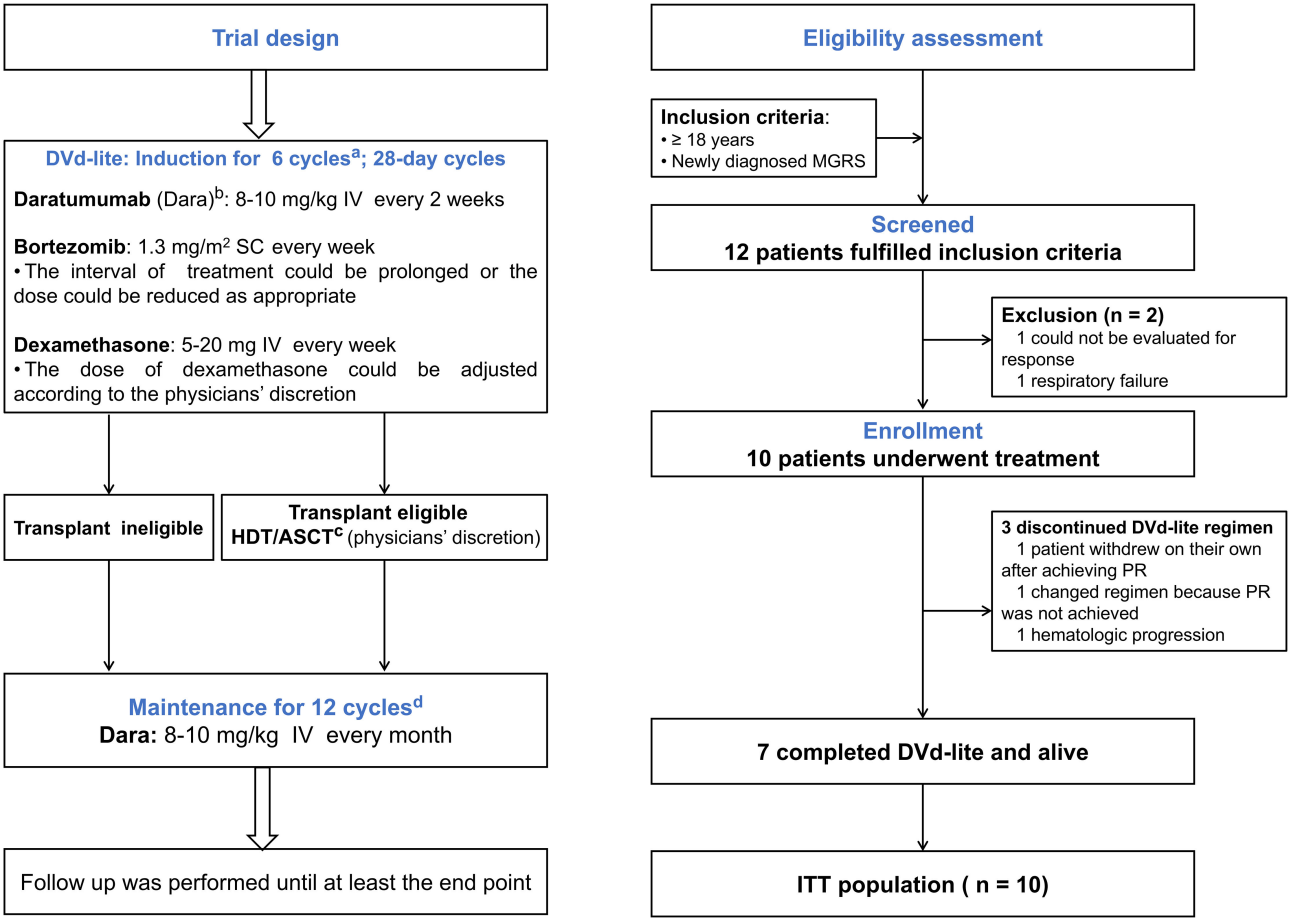
The trial design (left panel) and flow chart of the study (right panel). ^a^There were six cycles of induction therapy. ^b^Dara dose escalation was optional for patients failing achieving a partial response (PR) after one cycle or a very good partial response (VGPR) after three cycles. ^c^After 4–6 cycles of treatment, autologous stem cell transplantation (ASCT) consolidation therapy could be selected. ^d^Maintenance therapy was initially planned for 12 cycles, but the maintenance therapy duration among enrolled patients varied due to differences in treatment compliance and funding availability. Abbreviations: Dara, Daratumumab; IV, intravenously; SC, subcutaneously; HDT, high-dose therapy; ASCT, autologous stem cell transplantation; ITT population: intention-to-treat population.

As shown in **Figure 1** (left panel), this study enrolled patients who received six cycles of the DVd-lite regimen as induction therapy. Dara dose escalation was optional if patients did not achieve partial response (PR) after one cycle or did not achieve very good partial response (VGPR) after three cycles. For patients over 70 years old or with poorly controlled diabetes, the dose of dexamethasone could be adjusted according to the physicians’ discretion. After 4–6 cycles of treatment, autologous stem cell transplantation (ASCT) consolidation therapy could be selected. If disease progression (PD) occurred, patients were withdrawn from the study and switched to alternative therapeutic regimens.

Eligible patients (see **Figure 1**, right panel) were at least 18 years old with newly diagnosed MGRS and met the diagnostic criteria proposed by the International Kidney and Monoclonal Gammopathy Research Group in 2017 (5). Patients were excluded if they presented with acute cardiopulmonary function failure, inability to assess hematological or renal response, diagnosis of a hematological malignancy, ultrasound evidence of obstructive kidney disease, or an Eastern Cooperative Oncology Group performance status exceeding 3.

The primary endpoint was hematological overall response rate (ORR) during induction therapy, assessed in the intention-to-treat (ITT) population. The ORR is defined as the proportion of patients in the ITT population who achieved partial response (PR) or better. Secondary endpoints included kidney ORR (kidORR) and adverse events (AEs). Hematological and renal responses were primarily evaluated according to the response criteria for immunoglobulin light chain amyloidosis (16) (see **Supplementary Table 1** for specific criteria). In addition to clone-directed therapy, we also provided adequate renal supportive therapy, with details on the specific medications shown in **Supplementary Table 2**. AEs were reported according to Common Terminology Criteria for Adverse Events (CTCAE) version 5.0.

Descriptive statistics were summarized using frequencies and proportions for categorical variables. The number and percentage of response cases (PR or better) were calculated, with the denominator being the total number of subjects in the analysis population. The renal duration of response (DOR) is defined as the time from the first occurrence of response (PR or better) to the first occurrence of PD or death, whichever occurs first.

## 3 Results

### 3.1 Patients’ baseline characteristics

Between February 2024 and August 2025, ten patients were enrolled. The detailed demographic and clinical characteristics are presented in **Table 1**. Briefly, four patients had AL amyloidosis; three had monoclonal immunoglobulin deposition disease (MIDD), including two with LCDD; two had PGNMID; and one had light chain proximal tubulopathy (LCPT). The median age at enrollment was 52 years (range: 46-70). Seven patients were male (70%). The median bone marrow infiltration was 4.5% (range: 0%–11%). All those with heavy chain M protein were the IgG type (80%), and half of the ten patients had λ light chain M protein. The median level of M protein was 6.0 g/L (range: 0-22.7). Nine patients were M protein-positive as detected by immunofixation electrophoresis, and another patient was M protein-negative but had detectable clonal plasma cells in bone marrow and abnormal serum free light chain (FLC) ratio. The median values of involved FLC (iFLC) and the difference between involved and uninvolved FLC (dFLC) were 168.3 mg/L (range: 21.0-1715.8) and 147.2 mg/L (range: 6.9-1709.9), respectively. Regarding renal function, the median baseline estimated glomerular filtration rate (eGFR) was 78.7 ml/min/1.73m^2^, with six patients (60%) having eGFR less than 90 ml/min/1.73m^2^, while one patient was on dialysis at the initiation of treatment. Additionally, six patients had oedema/effusions. Diabetic nephropathy was observed in Patient 1 and Patient 9. Neurological symptoms were observed in Patient 6, while Patient 4 and Patient 10 had cardiac involvement.

**Table 1:** Patients’ demographic and clinical characteristics.

Patient number	Age(years)/ gender	Bone marrow plasma cells	M protein type	Serum M protein quantity (g/L)	Serum iFLC/dFLC (mg/L)	Hemoglobin (g/L)	Renal histology	eGFR (ml/min/1.73m2)	Proteinuria (g/24h)	UACR (mg/g•Cr)	NT-proBNP (pg/ml)
01	52/male	11%	IgG k	7.1	1227.1/1206.8	155	MIDD	112.5	4.71	7858.2	< 100
02	57/male	2.0%	IgG λ	4.1	47.1/7.8	123	PGNMID	66.0	7.02	2853.6	< 100
03	46/male	6.5%	IgG λ	22.7	184.3/174.8	138	AL	117.6	4.3	2241.4	< 100
04	58/female	4.5%	λ	1.0	1715.8/1709.9	110	AL	226.4	8.47	647.0	1200
05	60/male	0.0%	IgG k	6.4	21.0/6.9	95	PGNMID	73.0	1.94	1626.2	< 100
06	46/male	9.5%	IgG k and λ biclonal	IgG k (3.6)	90.7/72.4	120	AL	298.3	1.41	877.2	< 100
07	70/male	0.5%	IgG λ	7.2	483.7/463.8	126	AL	81.9	3.1	3016.8	< 100
08	51/male	0.5%	Negative	0.0	145.0/49.7	141	LCPT	75.4	1.1	141.8	< 100
09	49/female	4.5%	IgG k	5.8	152.2/118.6	83	LCDD	19.6	2.9	2430.0	< 100
10	52/female	7.0%	IgG k	6.1	742.8/707.5	63	LCDD	16.7	3.1	4002.5	18000

M-protein, Monoclonal immunoglobulin; iFLC, involvement of free light chain; dFLC, difference FLC; eGFR, estimated glomerular filtration rate; UACR, Urine albumin-to-creatinine ratio; NT-proBNP, N-terminal pro-B-type natriuretic peptide; MIDD, monoclonal immunoglobulin deposition disease; PGNMID, Proliferative glomerulonephritis with monoclonal immunoglobulin deposits; AL, light chain amyloidosis; LCPT, Light chain proximal tubulopathy; LCDD, light chain deposition disease.

### 3.2 Hematological response

**Figure 2:**
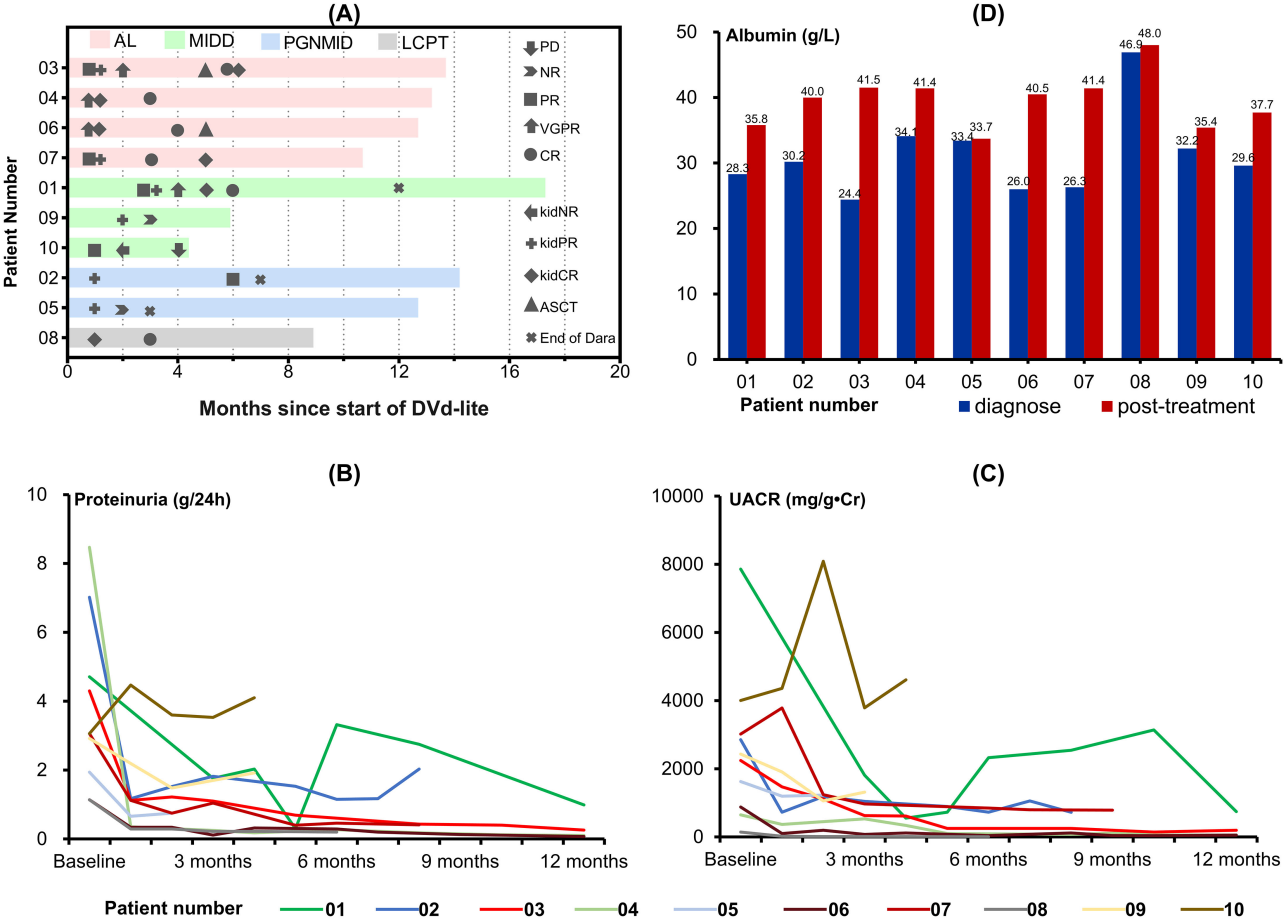
**(A)** Swimmer’s plots for the ten MGRS patients treated with DVd-lite regimen. Events are denoted in the figure legend. **(B)** Proteinuria and **(C)** UACR response from baseline to 12 months in patients who received at least two cycles of the DVd-lite regimen. **(D)** Changes in serum albumin levels of patients before and after treatment. PD, disease progression; NR, no response; PR, partial response; VGPR, very good partial response; CR, complete response; kidNR, kidney NR; kidPR, kidney PR; kidCR, kidney CR; ASCT, autologous stem cell transplantation. Patient 2 and 5 were evaluated using M-protein levels due to baseline dFLC levels below 20 mg/L.

The median follow-up of the cohort was 13.1 months (range: 4.7-17.6), and all ten patients were still alive at the end of the follow-up period. Eight out of the ten patients with evaluable disease exhibited hematological responses to the DVd-lite regimen (see **Figure 2A**). The hematological ORR was 80%, including 20% (2/10) reaching PR and 60% (6/10) achieving complete response (CR). Specifically, four patients with AL amyloidosis (including one who achieved CR after ASCT), one patient with MIDD, and one patient with LCPT achieved CR. The median number of treatment cycles for the eight patients who achieved the best hematological response was 3.5 cycles (range 1-6). Note that Dara dose was increased for Patient 10 because this patient achieved PR after one cycle but did not achieve very good partial response (VGPR) after three cycles. Unfortunately, PD still occurred after increasing the dose of Dara. The two patients who failed to achieve hematological response attained kidney PR (kidPR) following two treatment cycles. Of these, Patient 5 discontinued further therapy; Patient 9 switched regimens.

### 3.3 Renal response

All ten patients could be evaluated for renal response (see **Figure 2A**). The kidORR was 90%, including 30% (3/10) achieving kidPR and 60% (6/10) reaching kidney CR (kidCR). The median number of treatment cycles from diagnosis to best kidney response for the nine patients was 2 cycles (range: 1-5), and the median renal DOR was 9.6 months (range: 0.9-13.0). The dynamic changes in 24-hour proteinuria quantification and urine albumin-to-creatinine ratio (UACR) of all the patients are shown in **Figures 2B, C**, respectively. The median values of 24-hour urinary protein and UACR for the patients decreased from 3.1 g/24 h (range: 1.1-8.5 g/24 h) and 2335.7 mg/g•Cr (range: 141.8-7858.2 mg/g•Cr) at the time of diagnosis to 0.6 g/24 h (range: 0.1-4.1 g/24 h) and 457.8 mg/g•Cr (range: 6.4-3787.1 mg/g•Cr) at the last follow-up, respectively.

### 3.4 Other clinical response

The median serum albumin level among the ten patients increased from 29.9 g/L (range: 24.4-46.9) at diagnosis to 37.9 g/L (range: 33.7-41.5) at the last follow-up (see **Figure 2D**). Patient 6, who was found to have a double-clone M protein, presented with obvious peripheral nerve damage and severe diarrhea at the initial diagnosis, but all symptoms were effectively alleviated after six treatment cycles. In addition, Patient 10 exhibited a notable reduction in NT-proBNP levels, declining from 18000 pg/ml to 5800 pg/ml after four treatment cycles. Concurrently, her hemoglobin level improved from 63 g/L to 117 g/L.

### 3.5 Safety

No treatment-related deaths occurred. Most treatment-related adverse events (TRAEs) were grade 1-2, with only one grade 3 TRAE observed. This patient developed an allergic reaction during the initial infusion of Dara, manifested as mild dyspnea and bronchospasm, and was safely re-infused with Dara after active symptomatic management. All TRAEs are listed in **Table 2**. Infusion reactions (70%) were the most common AEs. Other AEs included peripheral neuropathy (40%, one patient discontinued bortezomib at the fifth cycle), constipation (30%), upper respiratory infection (20%), and herpes zoster (20%), among others.

**Table 2:** Summary of TRAEs in all the patients (n = 10).

Events	Event frequency (%)
Infusion reaction	7 (70%)
Peripheral neuropathy	4 (40%)
Constipation	3 (30%)
Upper respiratory infection	2 (20%)
Herpes zoster	2 (20%)
abdominal distension	1 (10%)
Diarrhea	1 (10%)
Bronchospasm caused by an allergy	1 (10%)
Pustular rash	1 (10%)
Muscle pain	1 (10%)
Facial folliculitis	1 (10%)
Lips ulcer	1 (10%)

## 4 Discussion

Current clinical management of MGRS remains heterogeneous, with limited standardized treatment protocols (17, 18). To the best of our knowledge, our study is the first prospective evaluation of the DVd-lite regimen as first-line therapy for MGRS patients. The results showed an 80% hematological ORR, which was numerically higher than that reported in previous retrospective studies on MGRS (11, 18, 19). Notably, all four AL amyloidosis patients achieved CR, possibly suggesting that the DVd-lite regimen could be more effective in this subgroup. Furthermore, this regimen has shown favorable tolerability. In particular, compared to the full-dose Dara regimen (20, 21), it reduced the incidence of infections, with no grade 3 or higher infections observed. Meanwhile, there was no obvious increase in the burden on heart and kidneys. These results indicate that the DVd-lite regimen is a feasible treatment option for MGRS, demonstrating significantly improved tolerability while maintaining hematological efficacy.

In some cases, hematological abnormalities of MGRS might be subtle or even absent, with no circulating paraprotein or light chains (7). This poses a challenge for the hematologist and the nephrologist alike as to how to assess treatment response. Consistent with this pattern, our study observed 90% (9/10) of cases with M protein below 10 g/L, and the initial dFLC levels of two patients with PGNMID (Patient 2 and 5) were even below 20 mg/L. In this clinical context, even if the M protein level is below 10 g/L, it can still be used to evaluate the hematological response. At the same time, therapeutic decisions should also take into account renal parameters, as these patterns are frequently observed in MGRS patients (7, 10, 22). Thus, renal response was chosen as the secondary endpoint in this study, and the cohort showed a kidORR of 90% with DVd-lite treatment. Meanwhile, both median 24-hour urine protein and UACR levels exhibited significant reductions, maintaining similar trajectories throughout the treatment period. These findings might also suggest that UACR could represent a more practical surrogate indicator than 24-hour urine collection for patients who are not suitable for collecting 24-hour urine samples. However, a larger sample size is still needed to determine the cut-off value for using UACR to evaluate the renal response of MGRS. Additionally, it is worth noting that for patients with MGRS, particularly those with comorbidities such as diabetes mellitus and hypertension, renal supportive care, including RAAS blockade, SGLT2 inhibitors, as well as glycemic and blood pressure control, among others, is also crucial for improving renal outcomes, in addition to clone-directed therapy.

In addition to hematological and renal responses, we also observed a series of other clinical improvements. The albumin level of this cohort significantly increased, rising from 29.9 g/L at the initial diagnosis to 37.9 g/L at the last follow-up, which was closely related to the improvement of the patients’ edema/effusion symptoms. Furthermore, the significant reduction in NT-proBNP levels in the two patients, the improvement in neurological symptoms and diarrhea in one patient, and the recovery of hemoglobin in two patients-all these improvements in clinical symptoms could significantly enhance the quality of life of the patients. These findings suggest that the DVd-lite regimen could lead to symptom improvement not only in the hematology and kidney, but also in other organs.

In summary, although our study cohort is currently limited and these promising results warrant further validation in a larger cohort, these initial findings provide proof-of-principle that the DVd-lite regimen could produce significant hematological and renal responses in patients with untreated MGRS, while maintaining favorable tolerability. Therefore, the DVd-lite regimen is demonstrated to be an effective and low-toxicity option for MGRS patients.

## Data Availability

The original contributions presented in the study are included in the article/**Supplementary Material**. Further inquiries can be directed to the corresponding authors.
