# Supplementary material for: Efficacy and safety of reduced-dose daratumumab plus bortezomib and dexamethasone (DVd-lite) in newly diagnosed MGRS patients
Source: Front Immunol. 2026 Jun 23;17:1722204. doi: 10.3389/fimmu.2026.1722204 (PMC13337708; doi:10.3389/fimmu.2026.1722204)
Supplement: Supplementary file 1 [file Table1.docx]

**Supplemental table 1**. Hematological and renal response criteria.

| **Hematological response** | **Criteria** |
| --- | --- |
| Complete response (CR) | Negative in serum IFE and normalization of serum FLC levels |
| Very good partial response (VGPR) | • Baseline dFLC ≥ 50 mg/L: reduction in dFLC < 40 mg/L  • Baseline dFLC < 50 mg/L: ≥ 90% reduction in serum M-protein |
| Partial response (PR) | • Baseline dFLC ≥ 50 mg/L: a greater than 50% reduction in the  dFLC  • Baseline dFLC < 50 mg/L: ≥ 50% reduction in serum M-protein |
| No response (NR) | Less than a PR |
| Progression | • From CR, abnormal FLC ratio (FLC must double) • From any response, 50% increase in serum M-protein to > 0.5 g/dL (a visible peak must be present) • Involved FLC increase of 50% to > 100 mg/L |
| **Renal response** | **Criteria** |
| Kidney CR (kidCR) | proteinuria ≤ 500 mg/24h and < 15% decline in baseline eGFR |
| Kidney PR (kidPR) | 31%-60% reduction in 24-hour proteinuria and a reduction in baseline eGFR of less than 30% |
| Kidney NR (kidNR) | Less than a kidPR |
| Kidney progression | ≥ 25% decrease in eGFR |

**Note.** Hematologic response could not be evaluated if dFLC < 20 mg/L and M-protein < 0.5 g/L; Renal response could not be evaluated if 24-hour proteinuria was less than 500 mg.
