# Supplementary material for: Efficacy and safety of reduced-dose daratumumab plus bortezomib and dexamethasone (DVd-lite) in newly diagnosed MGRS patients
Source: Front Immunol. 2026 Jun 23;17:1722204. doi: 10.3389/fimmu.2026.1722204 (PMC13337708; doi:10.3389/fimmu.2026.1722204)
Supplement: Supplementary file 2 [file Table2.docx]

**Supplemental table 2**. Background renal medications and supportive care of the ten patients.

| Patient Number | RAAS blockade | SGLT2 inhibitors | Other  antihypertensive drugs | Diuretics | Glycemic control | Roxadustat |
| --- | --- | --- | --- | --- | --- | --- |
| 01 |  | √ |  | √ | √ |  |
| 02 | √ |  | √ | √ |  |  |
| 03 |  |  |  | √ |  |  |
| 04 |  |  |  |  |  |  |
| 05 |  |  | √ | √ |  |  |
| 06 | √ |  |  | √ |  |  |
| 07 |  |  |  | √ |  |  |
| 08 |  |  |  |  |  |  |
| 09 | √ | √ | √ | √ | √ | √ |
| 10 | √ |  | √ | √ |  | √ |
